# Lack of recognition and documentation of stress hyperglycemia is a disruptor of optimal continuity of care

**DOI:** 10.1038/s41598-021-89945-3

**Published:** 2021-06-01

**Authors:** Eric D. Moyer, Erik B. Lehman, Matthew D. Bolton, Jennifer Goldstein, Ariana R. Pichardo-Lowden

**Affiliations:** 1 Penn State College of Medicine, 700 HMC Crescent Road, Hershey, PA 17033 USA; 2 Department of Public Health Sciences, Penn State College of Medicine, 90 Hope Drive, Suite 3400, Hershey, PA 17033 USA; 3 Information Services, Penn State Health and Penn State College of Medicine, Room 3315, 100 Crystal A Drive, Hershey, PA 17033 USA; 4 Department of Medicine, Milton S. Hershey Medical Center, Penn State Health, Penn State College of Medicine, Penn State University, 500 University Drive, Hershey, PA 17033 USA

**Keywords:** Type 2 diabetes, Risk factors, Preventive medicine, Metabolic disorders

## Abstract

Stress hyperglycemia (SH) is a manifestation of altered glucose metabolism in acutely ill patients which worsens outcomes and may represent a risk factor for diabetes. Continuity of care can assess this risk, which depends on quality of hospital clinical documentation. We aimed to determine the incidence of SH and documentation tendencies in hospital discharge summaries and continuity notes. We retrospectively examined diagnoses during a 12-months period. A 3-months representative sample of discharge summaries and continuity clinic notes underwent manual abstraction. Over 12-months, 495 admissions had ≥ 2 blood glucose measurements ≥ 10 mmol/L (180 mg/dL), which provided a SH incidence of 3.3%. Considering other glucose states suggestive of SH, records showing ≥ 4 blood glucose measurements ≥ 7.8 mmol/L (140 mg/dL) totaled 521 admissions. The entire 3-months subset of 124 records lacked the diagnosis SH documentation in discharge summaries. Only two (1.6%) records documented SH in the narrative of hospital summaries. Documentation or assessment of SH was absent in all ambulatory continuity notes. Lack of documentation of SH contributes to lack of follow-up after discharge, representing a disruptor of optimal care. Activities focused on improving quality of hospital documentation need to be integral to the education and competency of providers within accountable health systems.

## Introduction

Stress hyperglycemia is commonly seen in hospitalized patients. There are glycemic and metabolic changes derived from the stress response that accompanies medical procedures and acute illnesses, leading to myriad risks of potential complications^1^. Patients with hyperglycemia in the hospital can be classified into three different categories: known diabetes, undiagnosed diabetes, or true stress hyperglycemia. Stress hyperglycemia is defined as a transient elevation in blood glucose (BG) levels during acute illness or following invasive procedures experienced by patients without diabetes^2^. It is estimated that 11–12% of hospitalized patients develop stress hyperglycemia, with a linear increase in incidence with advanced age^1,3,4^. This incidence is considerably higher at about 23.9% in critically ill patients admitted to an ICU^5^, and reported in approximately 80% of cardiac surgical patients^6^.

Pathophysiologic changes involving glucose metabolism occur as a result of stress of illness. An increase in sympathetic stimulation secondary to acute illness, surgery, or trauma causes the release of catecholamines, cortisol, growth hormone, and other counterregulatory hormones that contribute to increased insulin resistance and subsequent elevations of BG concentrations^2,7^. This acute rise in BG can exacerbate oxidative stress, alter lipid and carbohydrate metabolism, and disrupt the interplay between immune, endocrine, and neural systems^8^. Disruption of these integral pathways is associated with an increased risk of infectious complications^9–13^, mortality in intensive and non-intensive care patients^1,11,14–19^, and extended hospital stays^11–13,16,18,20^. These risks seem to be higher in patients without a previous diagnosis of diabetes^1,18,21–27^.

Stress hyperglycemia appears to signal a disturbance in glucose metabolism that may predict a risk of progression to prediabetes and type 2 diabetes^5,28–31^. This risk seems to increase in a linear fashion that correlates with admission BG level and may lead to a diagnosis of diabetes within 3 years from hospital discharge in 15% of subjects^30^. Since those patients who develop hyperglycemia in the hospital will need continuity care, it is important to determine whether the glucose abnormality represents underlying diabetes or stress hyperglycemia^6,32–35^.

Patients benefit from the diagnostic characterization of their glucose abnormality, optimal management, accurate documentation, and cohesive continuity of care to help reduce morbidity risks associated with stress hyperglycemia^35^. Historically, clinical practice has been flawed by laxity concerning diabetes management in the inpatient setting^36^. Hyperglycemia is frequently viewed as a secondary concern, a non-consequential manifestation of acute illness, or a state that will subside upon the resolution of illness. These views potentially allow type 2 diabetes to remain undiagnosed and the conduct of a proper risk assessment, either in the hospital or subsequent to inpatient care to be delayed^34,36,37^.

Communication from the inpatient to the outpatient setting is typically accomplished through discharge summaries that provide relevant information regarding the hospital course. Detailed documentation of pertinent clinical events during hospitalization is often the preamble to comprehensive continuity of care necessary to help reduce risks of disease progression or deterioration, and poor outcomes. However, effective documentation of stress hyperglycemia during hospitalization is not a common practice which can lead to inadequate ambulatory follow-up care.

The consistency of acknowledgement and documentation of stress hyperglycemia, or how glycemic control is followed after hospital discharge is poorly understood. It is possible that the lack of consensus regarding how to define stress hyperglycemia, based on glucose cutoffs that contribute to hospital outcomes and long term-risk, prevents a more proactive approach to assess, manage and follow these patients. Current clinical guidelines recognize hyperglycemia as BG levels ≥ 7.8 mmol/L (140 mg/dL) and assessment for predisposing causes of hyperglycemia and further testing for possible underlying diabetes should occur. Furthermore, treatment of hospitalized patients should occur if BG levels are persistently above 10 mmol/L (180 mg/dL) regardless of a diagnosis of diabetes to prevent complications associated with hyperglycemia^6,35^.

We propose that expanding our understanding of scenarios of stress hyperglycemia is essential to enable strategies that can promote awareness about dysglycemia, to recognize of diabetes risk, to adequately claim the complexity of care rendered during hospitalization, and to plan for continuity of care. The purpose of this study is to examine the incidence of stress hyperglycemia and the practice of documentation in the hospital and upon transition of care. We provide recommendations on interventions aiming to (1) improve discharge planning and the quality of discharge summaries and (2) promote continuity of outpatient evaluation to assess risk of, or possible diagnosis of type 2 diabetes in patients with a history of stress hyperglycemia.

## Methods

We examined incidence and documentation of stress hyperglycemia in the electronic health records (EHR) among hospitalized adults ≥ 18-years-old through the continuum of care in an academic medical center during a 12-months period. Documentation of stress hyperglycemia during hospitalization course, in discharge summary, and at subsequent ambulatory visits were reviewed from March 2018 to February 2019. Our methods capitalized on EHR’s clinical decision support tools (CDS) to recognize scenarios of stress hyperglycemia. CDS uses person-specific computable health information and intelligent filters and processes data, and applies knowledge in the right clinical context to facilitate decisions to enhance health care and improve health outcomes^38,39^. The tools we employed are described below.

1. An algorithmic workflow codified to match common data elements corresponding to glycemic data and patient characteristics was used to recognize stress hyperglycemia events among hospitalized patients in real time. The algorithm was instituted as part of a hospital-wide program evaluating clinical decision support in the EHR with the purpose of addressing gaps in glycemic care by providing practice recommendations^40^. For the purpose of this study, we defined stress hyperglycemia among hospitalized patients as two point-of-care BG levels ≥ 10 mmol/L (180 mg/dL) at least 3 h apart within a 36-h-period during an inpatient encounter without documentation or biochemical evidence of diabetes. Our definition of stress hyperglycemia was more strict than the recommended threshold for monitoring and treatment in guidelines of 7.8 mmol/L (140 mg/dL)^6,32,33^. We used this threshold to avoid equivocal attribution of stress hyperglycemia as its recognition was followed by real-time notifications to clinicians through an alert-based clinical decision support program^40^.
2. A case detection tool and registry identified patients meeting biochemical criteria for stress hyperglycemia and who were lacking documentation of it upon hospital discharge in the diagnosis or problem list based on ICD-10 code (“hyperglycemia, unspecified” -R73.9), SnoMed, or IMO codes. This design applied criteria to the SAP Business Objects software, which was programmed to query common data elements in the EHR and automatically populate the case registry. In this registry, the BG inclusion criteria based on point-of-care testing was expanded to a- ≥ 7.8 mmol/L (140 mg/dL) once in alignment with criteria for recognition of stress hyperglycemia and screening for diabetes; b- ≥ 7.8 mmol/L (140 mg/dL) at least 4 times, c ≥ 10 mmol/L (180 mg/dL) at least twice, or d ≥ 13.9 mmol/L (250 mg/dL) at least once any time during hospital stay representing the variety of glycemic scenarios that are often encountered in the hospital. This intended to provide a more ample assessment of the frequency of such scenarios. Exclusions included a diagnosis of diabetes, prediabetes, or hyperglycemia already documented in the hospital problem list; biochemical evidence of dysglycemia confirmed by HbA1c ≥ 5.7%, or the use of diabetes medications. These exclusions ensured reliable attribution of stress hyperglycemia to acute illness and not to preexisting abnormal glucose metabolism. Both the algorithmic workflows to recognize real-time events, and the case detection tool and registry represent forms of clinical decision support.
3. We also undertook a direct chart abstraction process which assessed documentation of stress hyperglycemia after hospital discharge. This manual appraisal of documentation of stress hyperglycemia in discharge summaries and follow-up ambulatory notes was conducted in records of patients following within our healthcare system. It was done using a representative sample of admissions corresponding to a 3-months period of the entire 12-months cohort. Records of patients who did not have follow-up care in our health care system after discharge could not be evaluated for continuity.

### Statistical analysis

All descriptive analyses were performed using SAS version 9.4 (SAS Institute, Cary, NC). The data were analyzed in three ways. First, an estimate of the incidence of stress hyperglycemia considering the number of patients with at least one event of stress hyperglycemia compared to the adult population without a clinical or biochemical diagnosis of diabetes admitted to an academic health center during a 12-months period. Second, rate of documentation of stress hyperglycemia in the EHR medical problem or diagnosis list upon hospital discharge according to the number of cases reported in the case registry. Third, rate of documentation of stress hyperglycemia in hospital notes and post-discharge ambulatory documents in a representative sample from the case registry.

### Ethical approval

This study was approved by The Penn State College of Medicine Institutional Review Board (IRB) as STUDY00003330. All methods were performed in accordance with the guidelines and regulations set forth by the institution. A waiver of consent was also approved by the Penn State Health, Milton S. Hershey Medical Center Institutional Review Board for this study since all data was deidentified, thus representing no more than minimal risk to the participants.

## Results

We present the incidence of stress hyperglycemia in the study cohort and show demographics and admissions characteristics of patients during the 12-months of the study period in Table 1. We found a total of 467 hospitalized adults who experienced stress hyperglycemia, defined as having ≥ 2 BG levels ≥ 10 mmol/L (180 mg/dL) within a 36 h period. This accounted for 506 total patient admissions with stress hyperglycemia, including readmissions. We used this glucose level criterion to unequivocally attribute stress hyperglycemia to levels clinically suitable for acute treatment in the hospital and to determine incidence. These cases were observed among a qualifying inpatient population without a diagnosis of diabetes of 15,078 subjects, resulting in an incidence of stress hyperglycemia of 3.1%.

**Table 1:** Events of stress hyperglycemia among hospitalized patients: demographics and admission characteristics.

Variable	Total ^a^ N = 467 patients (%) corresponding to 506 admissions
**Age in years**	
Mean age	64.2 (± 15.7)
18–35	28 (6.0)
36–55	96 (20.6)
56–75	230 (49.3)
≥ 76	113 (24.2)
**Gender**	
Female	203 (43.5%)
Male	264 (56.5%)
**Race**	
Asian	11 (2.4%)
African American	27 (5.8%)
Caucasian	401 (85.9%)
Other	24 (5.1%)
Unknown	4 (0.8%)
**Ethnicity-Hispanic**	
Yes	21 (4.5%)
No	440 (94.02%)
Unknown	6 (1.3%)
**Admitting service**	
Surgical (General surgery and specialties)	66 (14.1%)
Medical (Medicine, FCM^b^ and specialties)	400 (85.7%)
Unknown	1 (0.2%)

^a^Mean ± SD, N (%).

^b^Family and Community Medicine.

Records showing 4 or more BG values ≥ 7.8 mmol/L (140 mg/dL), denoting milder glucose abnormality but in alignment with a clinical indication for diabetes screening, were used to assess the rate of documentation. Documentation of stress hyperglycemia was absent in the problem list of 521 hospital discharges corresponding to 503 patients meeting point-of-care BG criteria for stress hyperglycemia. There was a greater frequency of stress hyperglycemia among subjects between the 3rd and 7th decades, and a more substantial proportion of admissions corresponded to surgical services. A larger frequency of stress hyperglycemia corresponded to lower degree of hyperglycemia, as shown in Table 2.

**Table 2:** Lack of documentation of stress hyperglycemia in the EHR problem list upon hospital discharge: demographics and admission characteristics.

Variable	Total^a^ N = 521 admissions (%) corresponding to 503 patients
**Age (years)**	
Mean age	58.8 (± 17.2)
18–35	64 (12.7%)
36–55	118 (23.5%)
56–75	239 (47.5%)
> 75	82 (16.3%)
**Gender**	
Female	240 (47.7%)
**Race**	
Asian	5 (1.0%)
African American	29 (5.8%)
Caucasian	28 (5.6%)
Other	441 (87.7%)
**Ethnicity-Hispanic**	
Yes	28 (5.6%)
No	474 (94.2%)
Unknown	1 (0.2%)
**Medical service**	
Surgical (General surgery and specialties)	319 (61.2%)
Medical (Medicine, FCM^b^ and specialties)	202 (38.8%)
**Point of care blood glucose evidence of stress hyperglycemia**	
BG ≥ 7.8 mmol/L (140 mg/dL) × 1	521 (100.0%)
BG ≥ 7.8 mmol/L (140 mg/dL) × 4	465 (89.3%)
BG ≥ 10 mmol/L (180 mg/dL) × 2	279 (53.6%)
BG > 13.9 mmol/L (250 mg/dL) × 1	107 (20.5%)

^a^Mean ± SD, N (%).

^b^Family and Community Medicine.

A 3-months representative sample of the entire cohort lacking recognition of stress hyperglycemia was identified using discrete data elements in the EHR upon hospital discharge. This was examined for any evidence of stress hyperglycemia annotated in the discharge summary and/or continuity care notes after discharge. This yielded 124 admissions corresponding to 119 patients. Patients’ demographics and admission characteristics are presented in Table 3. Notably, similar to the findings in Table 2, a larger proportion of subjects had been admitted to surgical services.

**Table 3:** Documentation of stress hyperglycemia in hospital and post-discharge ambulatory documents: demographics and admission characteristics.

Variable	Total ^a^ N = 119 patients (%) corresponding to 124 admissions
**Age (years)**	
Mean age	60 (± 17)
18–35	13 (10.9%)
36–55	28 (23.6%)
56–75	55 (46.2%)
≥ 76	23 (19.3%)
**Gender**	
Female	59 (49.6%)
**Race**	
White or Caucasian	104 (87.4%)
Black or African American	7 (5.9%)
Asian	1 (0.8%)
Other	7 (5.9%)
**Ethnicity-Hispanic**	
Yes	4 (3.4%)
**Admitting service**	
Surgical (General surgery and specialties)	73 (59%)
Medical (Medicine, FCM^b^ and specialties)	51 (41%)
**Continuity of care setting**	
Ambulatory note available	60 (48.4%)
**Point of care blood glucose evidence of stress hyperglycemia**	
BG ≥ 7.8 mmol/L (140 mg/dL) × 1	124 (100.0%)
BG ≥ 7.8 mmol/L (140 mg/dL) × 4	109 (87.9%)
BG ≥ 10 mmol/L (180 mg/dL) × 2	56 (45.2%)
BG > 13.9 mmol/L (250 mg/dL) × 1	21 (17.0%)

^a^N (%).

^b^Family and Community Medicine.

## Discussion

The incidence of stress hyperglycemia in our study represents levels of hyperglycemia at which consistent glucose monitoring and treatment is recommended. We showcase two important stages of clinical practice that present opportunities to acknowledge and to document stress hyperglycemia, which we believe can enhance acute management and continuity of care. Failure to properly document stress hyperglycemia has implications on preventive care, value-based care, and organizational accountability. All of these attributes are important domains of quality in health care.

### Incidence of stress hyperglycemia

The 3.1% incidence of stress hyperglycemia in our study cohort denotes unequivocally elevated glucose values at which providers should consider treatment. Our detection criteria sustained a hospital-wide clinical decision support program employing real-time notifications and provision of practice recommendations^40^. Studies have reported a greater incidence in adult patients admitted to various hospital settings^1,3^, due to more inclusive criteria of hyperglycemia compared to our inclusion criteria. Umpierrez et al.utilized a fasting BG value greater than 7.0 mmol/L (126 mg/dL) or two random BG values greater than 11.0 mmol/L (200 mg/dL) as their threshold which yielded an incidence of 12%^1^. Russo and colleagues utilized any plasma BG value greater than 7.8 mmol/L (140 mg/dL) during a hospital stay as their threshold revealing an incidence of 12.13%^3^. The exact glucose threshold at which stress hyperglycemia becomes a clinical concern or biochemically defines the condition is unclear. However, clinical practice guidelines suggest a threshold of 7.8 mmol/L (140 mg/dL) to consider the evaluation for underlying diabetes and to continue glucose monitoring^6,32,33^. These guidelines recommend maintaining BG levels between 7.8 mmol/L (140 mg/dL) and 10 mmol/L (180 mg/dL). Glucose levels over 10 mmol/L (180 mg/dL) are associated with an increased risk for mortality, prolonged ventilatory support, renal replacement therapy, hyperbilirubinemia, septicemia, prolonged use of antibiotics, and increased ICU length-of-stay^41,42^.

Hyperglycemia attributable to the stress of illness warrants proactive recognition by healthcare teams to address the biochemical disarray and enhance awareness of the possible risk for diabetes among populations. In 2018, the United States experienced over 36 million hospital admissions between community and academic centers^43^. Considering our conservative incidence of stress hyperglycemia of 3.1% and incidence reported high as 12%, stress hyperglycemia can be encountered ranging from 1.1 to 4.3 million admissions annually. Importantly, many of these patients may have undiagnosed diabetes or are potentially destined to progress to diabetes over the subsequent months to years. This is a staggering number considering studies reporting that patients with stress hyperglycemia have an increased risk of progressing to type 2 diabetes compared to normoglycemic counterparts (3.48 OR)^5^ and (1.91 h)^31^. Therefore, the recognition of stress hyperglycemia in the hospital presents an opportunity to provide not only acute management but also an immediate assessment of risk and subsequent monitoring in the outpatient setting. Treating stress hyperglycemia with insulin would commonly require a more compelling glycemic abnormality than the glucose level recommended for screening and documentation. Screening, documenting and treating stress hyperglycemia are distinct and important interrelated elements for adequate practice and continuity of care.

### Documentation of stress hyperglycemia

Benefits of thorough documentation include the assessment of glycemic abnormalities in the hospital plan of care, which can facilitate more comprehensive acute management and diabetes screening, proper attribution of a diagnosis for which services were rendered during hospitalization, and provision of valuable information to providers responsible for the continuity of care. In our study, we examined the frequency of cases discharged without a diagnosis of stress hyperglycemia, despite meeting biochemical criteria, revealing 521 missed opportunities. Acknowledgment of the condition can offer patients benefits that may otherwise be overlooked and can facilitate adherence to practice guidelines for monitoring and diagnostic evaluation in patients with stress hyperglycemia^6,35^.

Additionally, our analysis revealed that all 124 cases that underwent manual abstraction did not have stress hyperglycemia as a secondary diagnosis in the problem list of their hospital discharge summary, which is the primary means of communication in the transition of care to the continuity care team. Two (1.6%) subjects had hyperglycemia mentioned as part of the narrative section of the hospital course in the discharge summary. This is consistent with documentation rates previously reported in the literature of 5% to 24%^4,34,44^. Similar deficiencies in discharge summaries have been described in heart failure^45^, dialysis^46^, and stroke^47^ cases.

Nationally, discharge summaries are lacking in terms of timeliness, transmission, and content^45,48^. A cross-sectional survey among physicians indicated that documentation of discharge diagnosis, complications during hospital stay, active medical problems at discharge, and arranged medical follow-up increase the quality of the discharge summary^49^. Providers understand the purpose and importance of an inclusive discharge summary; however, there is reduced awareness and knowledge regarding the relevance of stress hyperglycemia as demonstrated by the low incidence of the diagnosis. This postulates the need for institutions to develop strategies to reduce shortfalls and implement processes that promote best practices.

Effective action at different levels is needed to convey to providers the relevance of stress hyperglycemia (Table 4). Establishing standards for documentation should begin early during residency training. The ACGME reinforces this idea by requiring competency in many milestones, including appropriate utilization and completion of health records (ICS3) milestone^50^. Several institutions have developed quality improvement projects to improve the standards and timeliness of discharge summaries, resulting in statistically significant improvements of multiple domains^51–53^. Academic and community medical institutions should consider adopting continuing medical education programs into their curriculum to promote more accurate and timely discharge summaries. It is expected that this practice if reinforced early, will continue as clinicians advance through training into practice and can enhance the accountability of timely diagnosis, proper documentation, and adequate continuity of care.

**Table 4:** Considerations to optimize documentation of stress hyperglycemia.

Familiarize physicians with frameworks for treatment and continuity of care recommended by CMS, NQF, ADA, and the TOCCC^35,56,59,60^
Facilitate resident and practitioner didactic sessions addressing common pitfalls in documentation, and methods to improve discharge summaries^61–63^
Incorporate the use of recognized and endorsed approaches in the form of scoring rubrics and standardized discharge summary templates during education seminars aimed at improving physician documentation^51,63,64^
Implement clinical decision support systems into EHRs to improve the recognition of stress hyperglycemia, facilitate diagnostic evaluation, optimize glycemic management, and improve communication directed to continuity care providers, thereby improving patient outcomes^65^
Implement clinical decision support tools to improve the discharge planning process and identify patients who need specialized follow-up care once discharged^66^

*CMS* Centers for Medicare and Medicaid Services, *NQF* National Quality Forum, *TOCCC* Transitions of Care Consensus Conference, *ADA* American Diabetes Association.

In light of our results reporting on the need for documentation in discharge summaries, we believe it is important to also highlight the presence of documented hyperglycemia and the treatment rendered within daily progress notes over the duration of the hospitalization in our patient cohort. Although our goal was not to analyze daily progress notes for recognition of stress hyperglycemia by the treatment teams, it is important to mention that hyperglycemia was in fact frequently documented in day-to-day inpatient notes; however, the diagnosis of stress hyperglycemia is not being incorporated into discharge summaries which is important for continuity of care.

### Transition to ambulatory care and follow-up for stress hyperglycemia

In our cohort of 60 (48.4%) patients receiving continuity of care within our health system, there was no indication that stress hyperglycemia was addressed or acknowledged during subsequent visits. Tamez-Pérez et al*.* reported a similar phenomenon*,* showing only 5% of patients who experienced true stress hyperglycemia received follow-up care for dysglycemia upon discharge^34^. More broadly speaking, large international studies found that discharge summaries are frequently missing valuable information, contributing to the sub-par quality of continuity care in up to one-fourth of patients^54^. Inadequate communication in transitions of care hinders continuity of care, leads to an increased rate of hospital readmission, more adverse events, and worse outcomes^55,56^. This supports the need for proactive measures to improve documentation quality, highlighting hospitals as essential constituents to the accountability in the continuum of care of patients with hyperglycemia.

We propose that the need for close follow-up of stress hyperglycemia arises from the reported progression to type 2 diabetes^5,28–31^. In the general population, the median time to diagnosis of type 2 diabetes is 2.4 years, and more than 7% continue to be undiagnosed for at least 7.5 years^57^. Population health efforts are needed to reduce the number of patients unaware of their condition or those at risk of progressing to diabetes, given the opportunity for early intervention. The American Diabetes Association (ADA) encourages appropriate continuity of care for anyone who develops dysglycemia during their hospital stay^35^. Therefore, inpatient healthcare teams are in a unique position to identify numerous patients at risk for diabetes, whose only manifestation may be stress hyperglycemia. Prompt recognition and documentation may facilitate risk assessment, early diagnosis, and adequate planning for continuity of care. Our case identification tool and registry enabled recognition of persons at risk for diabetes by recognizing stress hyperglycemia. The utilization of this type of resource facilitates the assessment of populations at risk and can be used to promote the quality of care processes. This aligns with the recommendation of endorsing learning health systems as one of the pillars for centers of excellence for diabetes care^58^.

Improving the transition of care process from inpatient to outpatient settings has become a priority for stakeholders, including the Centers for Medicare and Medicaid Services (CMS), the National Quality Forum (NQF), the Transitions of Care Consensus Conference (TOCCC), and the ADA, as explained in Table 5. Each of these entities^35,56,59,60^ has sought to create a set of standards that offer a framework for efficient and effective transitions of care. We propose that adopting these frameworks will enhance continuity of care and influence long term outcome related to diabetes prevention.

**Table 5:** Key standards to improve the transition of care from the hospital to the outpatient setting.

**CMS**^59^ Coordinating care transition services that begin no later than 24 h prior to discharge Facilitate timely interactions between patients and post-acute and outpatient providers Provide timely and culturally and linguistically competent post-discharge education to patients, so they understand potential additional health problems or a deteriorating condition
**NQF**^56^ Implement a healthcare home to serve as a portal for communication and promote continuous coordination for all services of care Develop a plan of care and follow-up, which includes follow-up tests, treatments, and additional services needed Make available plans of care to healthcare homes, ensuring other healthcare entities timely access to the plan of care Enable point of care access to comprehensive, relevant data in EHR’s such as checklists for care plans, test results, and problem lists Convey the discharge plan of care, including all pertinent elements such as clinical status, medical diagnoses, and treatments/procedures performed
**ADA**^35^ Tailor structured discharge plan individualized to patients, beginning at the time of admission, and modified as patient's needs change Coordinate the outpatient follow-up visit with the primary care provider, endocrinologist, or diabetes educator within 1-month of discharge Provide clear and concise communication with outpatient providers regarding the cause of hyperglycemia, recommended treatments, and related complications or comorbidities
**TOCCC**^60^ Identify the minimum set of data to be relayed to the outpatient or follow-up care provider, including principal diagnosis and problem list Identify additional elements for the ideal transition of care to the outpatient facility, such as treatment and diagnostic plan, prognosis, and goals of care Arrange for communication and transmission of information from the inpatient to outpatient setting ideally upon the time of discharge Aligning timeliness of transmission of information with the urgency of follow-up required

## Conclusion

The results of our study reveal an opportunity for identification of patients at risk for diabetes considering their evidence of stress hyperglycemia during acute hospitalization. Lack of recognition of stress hyperglycemia otherwise obstructs proactively caring for the acute abnormality and effectively monitoring and addressing long-term hyperglycemia. Both scenarios have influence in the immediate and the subsequent outcomes of patients. We propose that activities focused on improving the quality of in-hospital documentation of stress hyperglycemia need to be an integral aspect of the education and the competency domains of providers promoted by accountable health care organizations.
